# Lunar synchrony, geography, and individual clocks shape autumn migration timing in an avian migrant

**DOI:** 10.1093/beheco/arae001

**Published:** 2024-01-17

**Authors:** Alicia M Korpach, Christina M Davy, Alex M Mills, Kevin C Fraser

**Affiliations:** Department of Biological Sciences, University of Manitoba, 50 Sifton Road, Winnipeg, Manitoba, R3T 2N2, Canada; Department of Biological Sciences, University of Manitoba, 50 Sifton Road, Winnipeg, Manitoba, R3T 2N2, Canada; Department of Biology, Carleton University, 1125 Colonel By Drive, Ottawa, Ontario, K1S 5B6, Canada; Department of Biology, York University, 4700 Keele Street, Toronto, Ontario, M3J 1P3, Canada; Department of Biological Sciences, University of Manitoba, 50 Sifton Road, Winnipeg, Manitoba, R3T 2N2, Canada

**Keywords:** migration phenology, internal clock, lunar synchrony, nightjar, Antrostomus, repeatability

## Abstract

Timing programs in animal migrants have been selected to synchronize movements that coincide with predictable resources on the breeding and nonbreeding grounds. Migrants face potential temporal conflicts if their migration schedules benefit from synchrony to conflicting rhythms associated with annual biogeographical (circannual) cues, lunar (circalunar) cues, or individually repeatable internal clocks. We repeat-tracked individuals of an avian lunaphilic species, Eastern Whip-poor-will (*Antrostomus vociferus*), for two to three successive autumn migrations to determine the influence of the lunar cycle, breeding location, and individual repeatability on migration timing. Almost all birds avoided departing for migration during a full moon, likely to take advantage of the bright moonlight to facilitate visual foraging and enhance pre-migration fattening. However, groups from two latitudinally distant sampling areas adjusted their autumn departure timing differently relative to the timing of the September full moon, presumably due to differences in seasonal prey availability. Individual repeatability increased throughout autumn migration, suggesting that the factors responsible for shaping migration timing may differ for different migration stages. Our results, that lunar synchrony, local climate, and individual internal clocks appeared to account for much of the variation in migration timing in whip-poor-wills, underscore the value of measuring potentially interacting factors that shape migratory behavior at species, group, and individual levels. It remains unclear if, or how, maintaining individually repeatable annual migration schedules provides an adaptive benefit for whip-poor-wills or other lunaphilic migrants. Further clarifying the reasons for phenotypic variation in whip-poor-will migration timing will improve predictions of their abilities to adjust migratory movements under changing environmental conditions.

## Teaser

Lunaphilic (moon-loving) birds have time-management challenges when they prepare for their long-distance migrations. We show that Eastern Whip-poor-wills avoid departing for migration during a full moon, likely to maximize moonlight-enhanced foraging benefits during this time. However, exactly when they leave also depends on where they live and the tendency for individual birds to have relatively consistent timing from one year to the next. Determining the relative influence of multiple factors on individuals, groups, and the entire species increases our understanding of the underlying causes of migration timing schedules and their potential flexibility.

## INTRODUCTION

Annual migration timing programs of long-distance migrants may have evolved to align with seasonal resources that support reproduction and survival at their breeding and nonbreeding locations, respectively (Lack 1954; Newton 2008). These programs require coupling between an endogenous mechanism that provides a framework for cyclical time-keeping (internal clock) and exogenous cues that ensure an organism’s schedule is appropriately tuned to its local environment (e.g., photoperiod) (Berthold 1984; Gwinner 1996; Karagicheva et al. 2016). Migrants also respond flexibly to unpredictable changes in local exogenous conditions. Thus, annual migration timing can vary widely among groups within a species (Conklin et al. 2010; Bulte and Bairlein 2013) and among individuals within a group (Stanley et al. 2012; Conklin et al. 2013). Migration synchrony, phenology, and consistency are characteristics of migration timing that shape population and community dynamics, gene flow, disease transmission, and individual fitness (Bauer et al. 2016). Therefore, quantifying how migration timing may vary across different biological levels of organization (e.g., species, groups, and individuals), and at various stages of migration, can clarify when and where migration timing is least flexible, and therefore, where we may expect environmental change to have the greatest impact.

Circannual cues that vary annually with geographic location (e.g., photoperiod, temperature, or precipitation) tend to be consistent predictors of environmental conditions on annual time scales and act as proxies for the availability of resources with expected fitness benefits, like food availability. For example, a certain temperature may trigger a behavior because that temperature is associated with peak prey abundance (Visser et al. 2006; McNamara et al. 2011). Avian migrants that use such predictable cues to tune their migration timing programs maximize the likelihood of a successful migration, or of reaching their migration endpoints when environmental conditions there are optimal for fitness (Visser et al. 2010; Winkler et al. 2014).

Some important environmental cues, however, vary on time scales that are independent of annual cycles. The circalunar cycle is highly predictable over a shorter time scale (the lunar month is 29.5 days), but full moons fall on different calendar dates each year. In many groups of animals, activity levels that match suitable moonlight levels have adaptive value by increasing nighttime foraging success, reducing predation risk, and synchronizing reproduction and communication (Mercier et al. 2011; Kronfeld-Schor et al. 2013; Prugh and Golden 2014). Potential responses to lunar cues during migration are rarely investigated but include increased or decreased migratory activity during specific lunar phases or illumination levels (Pyle et al. 1993; James et al. 2000; Meunier et al. 2008; Norevik et al. 2019). Responses to moonlight can be endogenous or exogenous. Given that tracking the moon can have fitness benefits, some species are predicted to have developed endogenous timing that accords with the reliability of the lunar cycle (Pinet et al. 2011; Andreatta and Tessmar-Raible 2020).

Although circannual or circalunar time-keeping behaviors are largely synchronized in species or groups, conspecifics can exhibit very different migration dates or durations (van Wijk et al. 2016; Neufeld et al. 2021). Behavioral differences among individuals can arise from genetic heritability or phenotypic plasticity (e.g., developmental or behavioral) (Piersma and Drent 2003), and individual personality traits may underlie movement strategies (Spiegel et al. 2017), leading to an array of migration tendencies within a population (Chapman et al. 2011). These individual variants of migration timing behaviors contribute to population-wide diversity and may be under selection pressure to allow populations to survive extreme events and to adapt to changing environmental conditions (Gill et al. 2014, 2019). The potential benefits of phenotypic variation in migration timing to the individuals themselves are less clear. In heterogenous environments, individual birds that match the timing of breeding with the timing of local food supplies have improved fitness (Thomas et al. 2001), and regulation of migration timing patterns that aid resource tracking should therefore also lead to improved fitness. The strength of individual repeatability in migration timing is species- and event-dependent (i.e., timing of departures or arrivals), but individual repeatability in avian migration events is common across landbirds, seabirds, and waterbirds (Holtmann et al. 2017; Franklin et al. 2022). Therefore, longitudinal studies that track the migration movements of individuals over consecutive years are critical to understand the causes and consequences of repeatable behavior and predicting how flexible species, groups, and individuals may be (Charmantier and Gienapp 2014).

**Figure 1:**
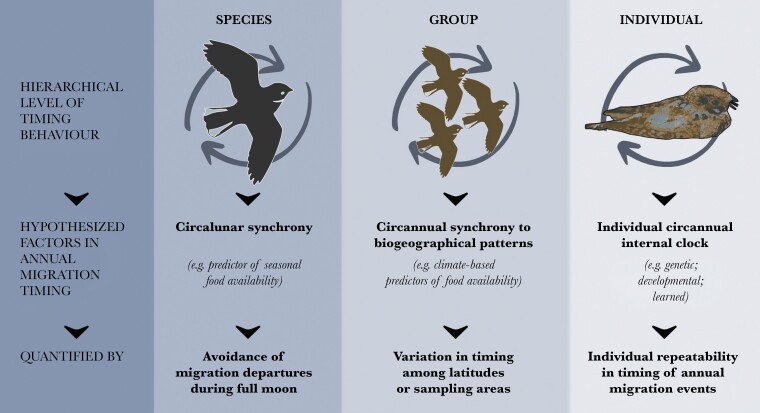
Factors hypothesized to explain annual migration timing in Eastern Whip-poor-wills (*Antrostromus vociferus*) at hierarchical levels of biological organization.

The goal of our study was to quantify factors that may shape annual migration timing programs at species, group, and individual levels (Figure 1). We used tracking data from a nocturnal, lunaphilic, bird from the nightjar family, Eastern Whip-poor-will (*Antrostomus vociferus*) (hereafter whip-poor-will). Whip-poor-wills are visual foragers that require some light to hunt their main prey of nocturnal moths. They forage most actively at dusk, at dawn, and during full moons when the sky is sufficiently lit (Cink et al. 2020). Whip-poor-wills breed widely in the eastern United States and southeastern Canada, and overwinter in Mexico and Central America. On the breeding grounds, their foraging and vocal activity are positively correlated with moonlight levels or moon altitude (Mills 1986; Knight et al. 2022). Temporal tracking of the lunar cycle to optimize foraging on moths during full moon periods may have a fitness benefit during breeding (Mills 1986; English et al. 2018). However, other considerations (e.g., rapidly deteriorating weather) could, at times, prevail over the benefits of waiting for the next full moon cycle (Mills 1986; English et al. 2018). The same should be true for migration timing; whip-poor-wills would benefit from using the moon for pre-migratory fattening to fuel migration, similar to other nightjar species (Norevik et al. 2019). However, it is unclear how individuals or groups of lunaphilic animals may have developed migration timing that integrates inter-annually varying cues such as moonlight with annually consistent ones associated with geography and individual clocks.

We hypothesized that three variables should act at the species, group, and individual levels to explain most variation in whip-poor-will autumn (southbound postbreeding) migration timing: 1) the timing of full moons, to which all whip-poor-wills should synchronize their foraging to prepare for and execute migration (species-level); 2) breeding location, which varies with latitude and other biogeoclimatic factors, and potentially reflects group-level circannual timing (group-level); and 3) individual internal clocks that establish repeatable annual migration timing for each bird (individual-level). We predicted that whip-poor-wills would avoid migrating during a full moon, and that the September full moon (which we consider the most influential moon in the migration departure period) and breeding location would explain most of the annual variation in autumn migration timing. We therefore also predicted that individual repeatability, while measurable, would contribute relatively less to annual migration timing events. To test our hypotheses, we GPS-tracked whip-poor-wills from two distant sampling areas located at different breeding latitudes, following individuals over two to three consecutive autumn migration cycles, with data collection occurring over 4 years to include a range of lunar cycle dates.

## MATERIALS AND METHODS

### Study area

**Figure 2:**
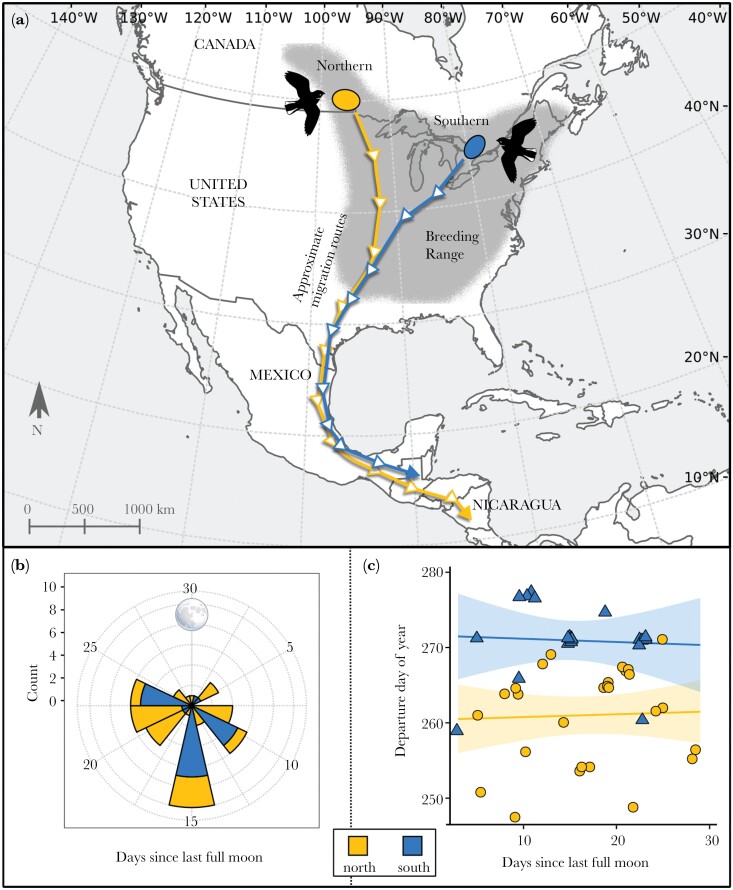
(a) Northern and southern sampling areas at the northern edge of the Eastern Whip-poor-will (*Antrostromus vociferus*) breeding range; (b) Number of autumn migration departures that occurred at different stages of the lunar month. Only a small proportion of birds departed in the days just before or just after a full moon. (c) There is no difference between the two sampling groups in the timing of autumn departures relative to lunar stage. Overlapping points are offset horizontally to improve visibility.

We studied migratory timing in whip-poor-wills departing from their breeding grounds, focusing on two sampling areas near the northern edge of the species’ range (Figure 2a). The sampling areas were ~1500 km apart and varied in latitude by ~5°/~500 km. The northern sampling area (latitude 49°–51°N, longitude 95°–98°W) spanned the Great Plains Parkland and Eastern Boreal Forest vegetation zones, and the southern sampling area (latitude 44°–46°N, longitude 77°–80°W) was in the Eastern Temperate Deciduous Forest zone (Baldwin et al. 2020). We refer to birds sampled in the two areas as belonging to two groups because whip-poor-wills are continuously distributed across their breeding range, and there currently is no evidence of genetically differentiated populations within their distribution (Cink et al. 2020). Whip-poor-wills from both groups overwinter in southern Mexico and Central America, with northern birds generally overwintering farther south (Figure 2a) (Korpach et al. 2022).

### Tag deployment and recovery of migration data

From May to July 2017–2020, we captured whip-poor-wills at the two sampling areas using song playback lures and mist nets. We focused on male whip-poor-wills because their territoriality increases capture and recapture probability, facilitating repeat annual tracking. We attached GPS tags (Pinpoint10 [1.5 g; Lotek Wireless Inc., Newmarket, Canada] or NanoFix Mini [2 g; PathTrack Ltd., Otley, UK]) to each bird with 0.75 mm Teflon cord leg-loop harnesses (Rappole and Tipton 1991). The mass of the tag did not exceed 3% of body weight. The tags were programmed to collect locations based on prior knowledge of the species’ residency dates on Canadian breeding grounds (English et al. 2017) and the life expectancy of the tags. Pathtrack tags began collecting daily locations within the first week of September, and Lotek tags began on either September 1, 15, or 25 (earlier dates were programmed for the northern sampling group). All tags stopped recording daily locations either on December 31, when the tag was full, or when the battery was depleted (approximately 2–4 months after recording began). We recaptured GPS-tagged birds on the breeding grounds after a full annual cycle to retrieve the tags and fit birds with new tags to allow repeat tracking (with the exception of the final year of the study).

Two additional birds were tagged with Motus radio tags (0.9 g; NanoTag model NTQB2-4-2S, Lotek Wireless, Inc, Newmarket, Ontario, Canada). These birds were not recaptured, and we monitored their departures via the existing Motus Wildlife Tracking System receiving station array (Taylor et al. 2017). The nearest Motus receivers in a migratory direction (south) of the tag deployment site were approximately 50–100 km away. We designated a departure date as the night that a bird was first detected by one of those receivers. The whip-poor-wills in our GPS dataset tended to make long initial migratory flights (>100 km), so it is reasonable to assume that we detected the Motus-tagged birds on their actual departure dates.

We recorded the following annual migration events for each individual: autumn migration departure dates, dates of crossing 35°N, dates of crossing 30°N, and winter site arrival dates. Migration departure and arrival dates are important milestones in a bird’s annual cycle, and measuring the timing of migration progression (i.e., crossing of mid-migratory latitudes) provides a fuller picture of migration timing. We chose latitudes 35°N and 30°N for statistical analyses because data from these locations yielded the largest sample sizes in our GPS dataset.

### Ethics approval

All work with wild birds was conducted under approval from the University of Manitoba Animal Care Committee (Animal Care Protocol Number F18 031/1(AC11388)); the Ontario Ministry of Mining, Northern Development, Natural Resource and Forestry Wildlife Animal Care Committee authorization #454; the Ontario Ministry of Natural Resources Wildlife Scientific Collector’s Authorization No. 1095705; the Manitoba Government Species at Risk Permits 18005 and 20007; and Manitoba Provincial Park Permits PP-PHQ-18-026, PP-PHQ-19-007, and PP-PHQ-20-003. Work in Algonquin Park was done with a Research Authorization from Ontario Parks. Bird capture and tagging were done under Environment and Climate Change Canada Banding Permit Nos. 10876F (AMK) and 10576 (AMM).

### Statistical analyses

Individual repeatability equations formed the foundation of our analyses that attribute variation in migration timing to either fixed factors (sampling area and lunar cycle) or random factors (individual birds). Repeatability, or intra-class correlation (ICC), is the proportion of variance in a dataset that is attributable to measurement error and phenotypic plasticity (Nakagawa and Schielzeth 2010), measured on a scale of 0 to 1. ICC is calculated by comparing the amount of variation within an individual’s repeated behaviors to the amount of variation among individuals in a population. Repeatability is high when individuals are highly consistent, relative to population-wide variation. *Adjusted* repeatability controls for the potentially confounding effect of covariates (fixed effects) and is commonly used to explore repeatability in animal behavior based on repeated measures of individuals (random effects). *Conditional* repeatability includes the fixed effect variance in the denominator of the repeatability equation and describes the phenotypic variance that is explained by fixed effects relative to the random effect.

We used the “*rptR*” package (Stoffel et al. 2017) in the software R (version 4.2.0) (R Core Team 2022) to fit linear mixed-effects models and estimate adjusted and conditional repeatability in the timing of annual migration events. Confidence intervals (95%) were estimated with 1000 bootstrap iterations. In all models, unless otherwise specified, the ordinal date (day of the year) was used as the response variable, and the individual bird was the random effect.

We also ran all models using lmer in the package “*lme4*” (Bates et al. 2015) to evaluate model performance more directly. We used “*MuMIn*” (Bartoń 2022) to calculate conditional *R*^2^ (variance explained by fixed and random effects), marginal *R*^2^ (variance explained by fixed effects), and AIC corrected for small samples (AIC_c_) (Hurvich and Tsai 1989) to compare the quality of the departure date models. We diagnosed simulated model residuals with “*DHARMa*” (Hartig 2021) and did not identify serious misspecification.

### Estimating effects of lunar cycle, sampling area, and individual repeatability on migration timing

We tested the full-moon foraging hypothesis in two ways. First, we calculated the frequency of autumn migration departures that occurred during a full moon. Moon brightness does not increase or decrease linearly throughout the lunar cycle. We restricted this test to the day of the full moon because moonlight, and therefore whip-poor-will activity, is disproportionately high on these nights, compared to even a few nights before or after the full moon. The peak expected migration departure period occurs in mid to late September at our study sites. Because full moons fall on different calendar dates each year, we used tracking data over several years to capture a range of full moon dates throughout the migration departure period. Second, we looked at the relationship between multi-day migration stopover durations and the lunar phase on the date each stopover was initiated. We defined a multi-day stopover site as a location where a bird did not move farther than 5 km in 24 h. Whip-poor-wills do not typically range farther than a few hundred meters once they arrive at a stopover or winter site, so these locations were clearly identifiable in the GPS tracks. For the stopover test, we only used data from birds that were tracked over their full migration from breeding to winter site.

We then built two linear models to test the predictions that lunar cycle and sampling area would explain most of the variation in migration timing: one with sampling area as the only covariate, to account for differing annual environmental conditions experienced by whip-poor-will groups, and another that included an interaction between sampling area and September full moon date. This interaction determined whether birds that may benefit from following consistent annual migration schedules, in accordance with their respective biogeographic regions, differ in how they synchronize their timing with an annually varying variable, the lunar cycle.

To test our assumption that the effect of the sampling area included latitudinal effects, we also modeled the exact latitude of each sample instead of the binary category of the sampling area. Latitude explained almost as much of the variation as the sampling area. However, the sampling area model fit better, as compared by AIC_c_ scores. We hereafter only refer to the sampling area model results.

Finally, to test our prediction of low individual repeatability in migration timing, we examined the proportion of variance (adjusted or conditional repeatability) explained by the fixed and random effects in the interaction model described above. Inaccuracy in either measure of within-individual consistency or population-level variation can lead to an unreliable ICC statistic. To increase the reliability of the repeatability estimate, we included additional single-tracked birds (1 year only) in the measurement of population-level variance (e.g., Fraser et al. 2019). We therefore included data from single-tracked birds in our main models but tested additional models only with repeat-tracked birds to ensure that our interpretation was not greatly altered (Supplementary Tables S1 and S2).

Additional intercept-only models to detect repeatability in individual responses to lunar cues other than a full moon (i.e., adherence to a circalunar schedule) did not converge, so we visually explored the absolute within-individual and population-level variation in lunar cue on each individual’s departure date. We visually examined the GPS tracking data to determine whether individuals reused stopover sites.

## RESULTS

### Migration data

We deployed 108 GPS tags and two Motus tags on 80 adult males (37 northern and 43 southern), placing 0, 13, 19, and 25 tags on northern birds and 10, 18, 27, and 0 tags on southern birds in the four successive years (numbers include tags from repeat-tracked individuals). We retrieved 63 GPS tags (57%) from 45 individuals (25 northern, 20 southern). GPS-tagged birds for which we recorded body mass had an average mass of 60.1 g at the first deployment (*N* = 59) and 61.8 g at the second deployment (*N* = 10), indicating that the GPS tags were not detrimental to the birds’ physical conditions. Not all retrieved tags contained usable data due to tag malfunctions or pre-programmed schedules that missed autumn departure or winter arrival dates.

Our complete tracking dataset contained sufficient data from up to 40 birds for analyses (Tables 1 and 2). We detected departure dates for the two Motus-tagged birds, but the Motus array was too sparse in the whip-poor-wills’ migratory range to detect the dates of crossing mid-migratory latitudes or winter arrivals. The GPS tags yielded 30 1-year tracks, 15 2-year tracks, and two 3-year tracks. Repeat track sample sizes (and number of repeat-tracked individuals) for each test were: autumn migration departure date, *N* = 9 (8); date of crossing 35°N, *N* = 15 (13); date of crossing 30°N, *N* = 12 (10); and winter-site arrival date, *N* = 5 (5).

**Table 1:** Adjusted repeatabilities (intra-class correlation [ICC], with standard error [SE] and confidence intervals [CI]) for the timing of Eastern Whip-poor-will migration events, after controlling for fixed effects

Migration event	Model	Tracks	Ind	Variation from random effect (ICC)	ICC SE	Lower ICC CI	Upper ICC CI	*P*
Autumn departure date	doy ~ 1 + (1|individual)	48	39	0.77	0.13	0.48	0.93	0.001
	doy ~ area + (1|individual)	48	39	0.63	0.18	0.22	0.9	0.003
	doy ~ area × SeptFullMoon + (1|individual)	48	39	0.64	0.15	0.36	0.94	0.002
Lat 35°N crossing	doy ~ area × SeptFullMoon + (1|individual)	55	40	0.6	0.15	0.29	0.88	0.005
Lat 30°N crossing	doy ~ area × SeptFullMoon + (1|individual)	51	39	0.67	0.14	0.37	0.93	0.01
Winter arrival date	doy ~ area × SeptFullMoon + (1|individual)	36	31	0.8	0.12	0.59	0.99	0.018

Competing models are only reported for autumn departure date. doy = ordinal day of year. area = sampling area. SeptFullMoon = date of September full moon. Tracks = total number of samples collected. Ind = total number of individuals tracked. Sample sizes include both single- and repeat-tracked birds.

**Table 2:** Conditional repeatabilities (intra-class correlation [ICC], with standard error [SE] and confidence intervals [CI]) for the timing of Eastern Whip-poor-will migration events and proportion of variation explained by random and fixed effects

Migration event	Model	Tracks	Ind	Variation from random effect (ICC)	ICC SE	Lower ICC CI	Upper ICC CI	*P*	Pseudo-*R*^2^ (marginal)	Pseudo-*R*^2^ (conditional)	AICc
Autumn departure date	doy ~ area + (1|individual)	48	39	0.35	0.13	0.07	0.61	0.003	0.44	0.79	299.6
	doy ~ area × SeptFullMoon + (1|individual)	48	39	0.31	0.11	0.13	0.55	0.002	0.52	0.83	299.3
Lat 35°N crossing	doy ~ area × SeptFullMoon + (1|individual)	55	40	0.42	0.13	0.18	0.69	0.005	0.3	0.72	---
Lat 30°N crossing	doy ~ area × SeptFullMoon + (1|individual)	51	39	0.51	0.13	0.25	0.76	0.001	0.26	0.75	---
Winter arrival date	doy ~ area × SeptFullMoon + (1|individual)	36	31	0.61	0.13	0.39	0.87	0.018	0.23	0.84	---

Competing models are only reported for autumn departure date. AICc for the null departure date model was 311.1. doy = ordinal day of year. area = sampling area. SeptFullMoon = date of September full moon. Tracks = total number of samples collected. Ind = total number of individuals tracked. Sample sizes include both single- and repeat-tracked birds.

### Effects of lunar cycle, sampling area, and individual repeatability on migration timing

September full moons occurred on 6 September 2017 (ordinal day of year (doy) 249), 24 September 2018 (doy 267), 13 September 2019 (doy 256), and 2 September 2020 (doy 246). Autumn departure dates (*N* = 46) ranged from 4 September (in 2018) to 4 October (in 2018) (mean 21 September ± 8 days standard deviation [SD]).

Almost all birds avoided initiating migration during a full moon (Figure 2b), even though birds from the two sampling areas departed at significantly different times, with northern birds leaving earlier (Figure 2c). Only one of 48 of autumn migration departures occurred during a full moon night, and three departures occurred within three days before or after a full moon (Figure 2b). The relationship between the departure date and the number of days since the last full moon did not differ between northern and southern sampling areas (Figure 2c). We recorded 179 multi-day stopovers from 35 birds with full migration tracks, with a mean duration of 6.9 days (±4.5 SD). The longest stopovers tended to be initiated when the moon phase was between 56% and 66% (i.e., in a 29.5-day lunar cycle beginning and ending at the new moon phase, 56% occurs just after a full moon) (Figure 4).

**Figure 3:**
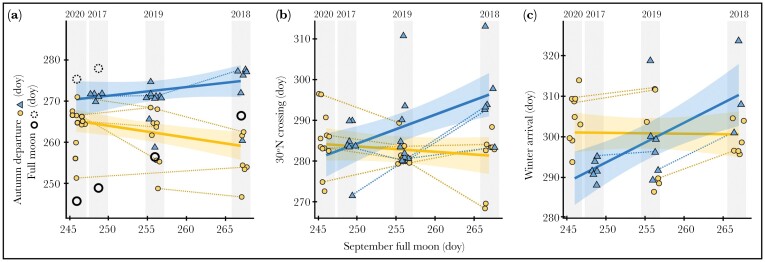
Eastern Whip-poor-wills (*Antrostromus vociferus*) from two breeding areas (northern = orange circles; southern = blue triangles) time migration departures to avoid a full moon, and exhibit individually repeatable migration timing (dashed lines connect individuals over two or more years of tracking. (A) When a September full moon (closed circles) occurs late in September, as in 2018, northern birds depart earlier and southern birds depart later than when a full moon falls early in the migration departure window, as in 2020 and 2017. In years with an early September full moon, birds also depart before the October full moon (dashed circles). (B) Group-level differences in migration timing decrease by mid-migration (results of crossing 35°N not plotted for simplicity). (C) Arrival at overwintering sites is similar between the groups. Annual migration dates (y-axes) are plotted against a single September full moon date (x-axes and shading centered on the September full moon date) but offset horizontally within the shaded bars to improve visibility. doy = ordinal day of year.

Although the sampling area explained most of the variation in autumn departure timing in the conditional repeatability model, the predictive ability of the model improved when we included an interaction with the September full moon date (Table 2). Simple slopes of the interaction effect indicated that northern and southern birds timed their migration differently with respect to the date of the September full moon (Supplementary Table 3). Birds in the northern group departed earlier than average in the year with the latest September full moon, but there was no association between the full moon date and their mid-migration and winter site arrival. Birds in the southern group departed later, reached mid-migration later, and arrived at their winter sites later in the year with the latest September full moon (Supplementary Table S3; Figure 3a–c)

After controlling for the potentially confounding effects of the sampling area and the timing of the September full moon, individual repeatability (ICC) in autumn migration departure dates was 0.64 and increased to 0.8 for winter site arrival (Table 1). When variation due to sampling area and September full moon date were included in the estimate, these effects explained a large proportion of the variation in migration timing (as seen by lower conditional ICC values [Table 2] compared to adjusted ICC values [Table 1]). The relative contribution of the fixed effects compared to the random effect (i.e., marginal *R*^2^ vs. ICC) decreased during the progression from autumn migration departure (0.52 vs. 0.31) to winter arrival dates (0.23 vs. 0.61) (Table 2).

Repeat-tracked individuals tended to consistently migrate earlier or later than the population average (dashed lines in Figure 3a–c): 6/8 birds for autumn departure and 4/5 birds for winter site arrival. Within-individual variability in autumn departure dates between the first and second year of tracking (Figure 3) ranged from 0–10 days (mean ± SD: 4.0 ± 3.4). Variation in the dates of crossing latitude 35°N was 2–17 days (7.8 ± 4.0), crossing latitude 30°N was 1–13 days (6.0 ± 4.3), and winter arrival was 1–10 days (mean 4.8 ± 3.7 SD).

We did not find evidence of individual repeatability reflecting potential tracking of various lunar cues, other than the full moon date, during autumn departures (Supplementary Figure S1). Repeat-tracked individuals followed similar routes, relative to variation exhibited within the northern and southern groups (Supplementary Figure S2), but none used the same stopover sites among years. The minimum distance between the two nearest stopover sites in an individual’s consecutive tracks was 11 km. In contrast, the winter site location was highly repeatable: 100% (*N* = 6) of birds that we repeat-tracked to their winter sites returned to the same locations, within a few hundred meters of the previous year’s winter site.

## DISCUSSION

Repeat-tracking of migratory individuals across a species’ range can reveal how different factors shape migration timing programs at the species, group, and individual levels. The early stage of autumn migration timing is strongly predicted in lunaphilic whip-poor-wills by synchrony with the lunar cycle (species-level) and geographic location (group-level)—biogeographic factors that shape departure timing from the temperate breeding grounds. Despite this species- and group-level synchrony, individual whip-poor-wills maintain remarkably repeatable individual timing among years (individual level).

### Effects of lunar cycle and sampling area on migration timing

Whip-poor-wills may enhance pre-migratory fattening by exploiting optimal foraging opportunities with a full moon during the time when prey is likely to be available. Our study was not designed to untangle the effects of potential heritable (genetic) variation between the two sampling areas from variation in environmental conditions (e.g., temperature or photoperiod), and further experiments would be required to do so (e.g., Partecke et al. 2004; Helm et al. 2009). However, our results imply that circannual, and possibly circalunar, rhythms are integrated to develop group level timing programs in whip-poor-wills. The interaction between the sampling area and the September full moon date revealed that although the whip-poor-wills from both areas did respond to a full moon during the autumn migration departure period, they responded differently. When the September full moon occurred later in the month, northern birds departed earlier, presumably to avoid flying during a full moon, whereas southern birds departed later (Figure 3a, Supplementary Table S3). This suggests that the northern birds’ response to the full moon may be anticipatory, which could stem from an endogenous circalunar rhythm cued in part to the moon phase. It also suggests that birds are adapted to waning prey availability in the autumn. Advancing their autumn departure timing in years when the September full moon falls late in the season allows the birds to reach migratory stopover locations where aerial insect prey are still active during the upcoming September full moon.

Our results tentatively suggest that whip-poor-will autumn departure timing partly reflects temperature-mediated prey availability. Latitudinal differences in migration timing occur in several species of aerial insectivores, including whip-poor-wills (Gow et al. 2019; Neufeld et al. 2021; Korpach et al. 2022). In temperate climates, latitude-related temperature changes may provide cues for birds to leave before foraging conditions deteriorate with the onset of winter. Whip-poor-wills from the northern sampling area would likely face below-freezing temperatures if they delayed their migration departure until October, while birds in the southern area would not (Environment and Climate Change Canada 2022a). Regional occurrence and abundance of whip-poor-wills are associated with the abundance of moths and beetles, particularly larger moth species (English, Nocera, et al. 2017; Souza-Cole et al. 2022), and most insects require air temperatures of 12–15 °C to initiate flight (Heath et al. 1971; Cox et al. 2007). Only a small number of cold-adapted Lepidopteran moth species can fly at near-freezing ambient temperatures (Heinrich 1987), and even where they co-occur with whip-poor-wills, they are unlikely to be sufficient prey for pre-migratory fattening.

**Figure 4:**
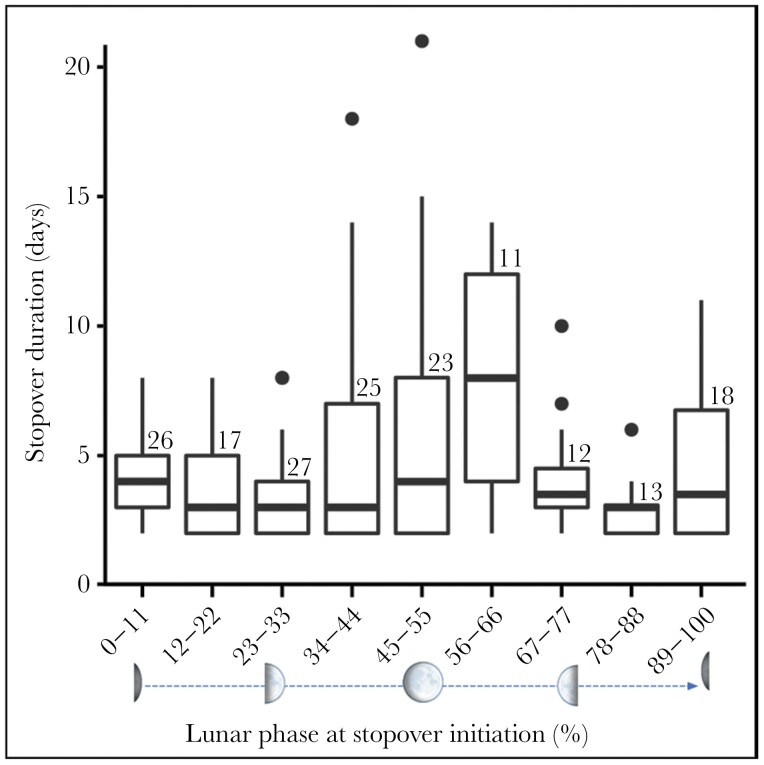
Durations of Eastern Whip-poor-will (*Antrostromus vociferus*) stopovers that were initiated during different phases of the lunar cycle (50% moon phase is a full moon). Longer stopovers tended to be initiated when the moon phase was between 56% and 66% (immediately following a full moon). Stopovers of one day in duration were excluded from the analysis. Numbers above the boxplots are sample sizes (numbers of stopovers) for each category of moon phase.

During migration, lunaphilic aerial insectivores would benefit from stopping to forage during a full moon, rather than making migratory flights, and we found that longer whip-poor-will stopovers were often initiated just after a full moon phase (Figure 4). However, stopover duration and lunar-mediated departure timing were not correlated in European nightjars (*Caprimulgus europaeus*) (Norevik et al. 2019), indicating that responses to the lunar cycle can vary even between species with similar ecologies. Some species or individuals may also avoid flying on nights with bright moonlight as a tactic to minimize predation risk or improve celestial navigation (Pyle et al. 1993; Kanda et al. 2016). High individual variation in migration timing in both European nightjars (Norevik et al. 2019) and whip-poor-wills (Tables 1 and 2) suggest that moonlight responses during migration may be species-, individual-, or context-dependent.

We focused our study on adult male whip-poor-wills because of sampling logistics, and we do not know how females or juveniles of this species may respond to the lunar cycle. Whip-poor-will chicks at the northern edge of the breeding range generally fledge by July (English et al. 2018; personal observation), so neither male nor female adults should be restricted by caring for young by the time they depart on migration in September. If a full moon optimizes foraging for all individuals, we expect that both adults and juveniles would take advantage of it, but this remains to be tested.

### The role of daily variation in biogeographic factors

Cyclical annual migration timing programs alone do not guarantee a successful migration because daily fluctuations in weather and food availability, and the animal’s internal state (e.g., fat stores, molt stage), affect how it prepares for each stage of migration (Gwinner 1996; Sjöberg et al. 2015; Stanley et al. 2022). Although we provide evidence for individual timing and responses to lunar and biogeographic cues, the small amount of remaining variation in the migration departure dates was likely a result of direct responses to daily weather and individual body conditions. Short-term temperature decreases may prompt early departures (Klinner and Schmaljohann 2020), although no large temperature anomalies occurred at our study sites that would likely result in the group-level variation that we observed (Environment and Climate Change Canada 2022a, b). Aerial insectivores may be unable to forage effectively during nights with high precipitation (Davy et al. 2022), and whip-poor-wills could respond opportunistically to unpredictable changes in moonlight availability due to cloud cover, as has been shown in other nocturnal species (Lockard 1975). The single breeding ground departure that occurred during a full moon in our study was on a night when the region had approximately 20 cm of daily rainfall (Environment and Climate Change Canada 2022b), and foraging conditions would have been poor. Provided that the individual had accumulated sufficient fuel reserves, and wind conditions were favorable (Weber et al. 1998; Liechti 2006), the trade-off may have been to depart for migration instead.

Predicting stochastic weather-mediated foraging activity and migration movements in whip-poor-wills is complicated by the fact that they have the physiology to cope with unfavorable conditions (low basal metabolic rate and the ability to enter daily torpor) (Lane, Brigham, et al. 2004; Lane, Swanson, et al. 2004), which should help to maintain consistent annual migration programs. Torpor use in the autumn may allow whip-poor-wills to complete fuel deposition in preparation for migration, especially if it is important that preparations coincide with an approaching full moon and that a individuals' departure dates follows predetermined schedules.

### Individual repeatability in migration timing

Migration timing phenotypes have a strong genetic basis, but it is likely that a combination of experience, condition, sociality, age, sex, local environment, and seasonal nesting dates contribute to an individual bird’s migration timing (Potti 1998; Pulido 2007; Liedvogel et al. 2011; Both et al. 2016; Bani Assadi et al. 2022; Loonstra et al. 2023). We cannot be sure of the suite of mechanisms underlying the variation in migration timing in our tracked whip-poor-wills, but we generally found high individual repeatability in autumn departure, mid-migration crossing, and winter arrival dates. Repeat-tracked whip-poor-wills in our study tended to maintain earlier or later migration timing than conspecifics, even when departing from adjacent breeding sites. Such phenotypic variation in migration timing may be advantageous in a relatively solitary species like the whip-poor-will. Although whip-poor-wills exhibit some breeding-winter migratory connectivity, individuals that breed close together can overwinter far from each other (Korpach et al. 2022; Skinner et al. 2022). Therefore, individuals would not benefit from synchronizing their migratory timing with conspecifics to optimize energy gain and use, aid navigation, or share information about foraging (Beauchamp 2011). Rather, variation in individual timings within a population may arise where optimal migration departure and arrival timing varies for each individual. High winter site fidelity (this study; Bakermans et al. 2022) may partially explain the high individual repeatability in the late stages of migration, particularly if the winter sites have predictable resource phenology. Familiarity and food predictability at nonbreeding locations remove uncertainty from migration and are expected to promote survival (Newton 2008; Cresswell 2014). Monitoring individual whip-poor-wills across the full annual cycle, including measures of reproductive success, physiological condition, resource availability, and competitive behavior in the nonbreeding season, would be required to understand the advantages or disadvantages of departing from a breeding site or arriving at a winter site at relatively consistent times each year.

Across avian taxa, autumn migration departure dates tend to be less repeatable than spring arrival or arrival to nonbreeding grounds (Conklin et al. 2013; Fraser et al. 2019; Franklin et al. 2022). Our data were consistent with this pattern, as individual repeatability increased from autumn departure to winter site arrival (absolute ICC 0.63–0.81 [Table 1] and conditional ICC 0.31–0.61 [Table 2]). Lower autumn departure repeatability can be due to lower urgency in post-breeding movements or varying outcomes of an individual’s annual breeding success (Phillips et al. 2005; Yamamoto et al. 2014). In our dataset, however, population-level variation may have driven the repeatability scores. The absolute within-individual variation in autumn departure and winter arrival dates were similar, but among-individual variation was lower in autumn departures than in winter site arrivals. Lower variation among individuals in autumn departure timing suggests that migration at that stage is controlled relatively strongly by biogeoclimatic factors that synchronize individuals. By late migration, individuals are less constrained by seasonal climatic factors experienced on the temperate breeding grounds, as well as by the need to maximize foraging during a full moon to prepare for a long migration. Our result that the relative contribution of the sampling area and September full moon decreased from autumn departure to winter arrival (marginal *R*^2^ 0.52–0.23, Table 2), also supports this interpretation. The whip-poor-wills in our study did not show fidelity to particular stopover locations during migration, suggesting that individuals could be able to partly compensate for strict timing programs by adjusting migration routes or daily flight distances.

## Supplementary Material

arae001_suppl_Supplementary_Tables_S1-S3_Figures_S1-S2

## Data Availability

Analyses reported in this article can be reproduced using the data provided by Korpach et al. (2023).
