# Supplementary material for: Lunar synchrony, geography, and individual clocks shape autumn migration timing in an avian migrant
Source: Behav Ecol. 2024 Jan 17;35(2):arae001. doi: 10.1093/beheco/arae001 (PMC11453104; doi:10.1093/beheco/arae001)
Supplement: arae001_suppl_Supplementary_Tables_S1-S3_Figures_S1-S2 [file arae001_suppl_supplementary_tables_s1-s3_figures_s1-s2.docx]

# Supplementary Information

**Lunar synchrony, geography, and individual clocks shape autumn migration timing in an avian migrant.**

Korpach, AM, Davy, CM, Mills, AM, and Fraser, KC. 2024. Behavioral Ecology.


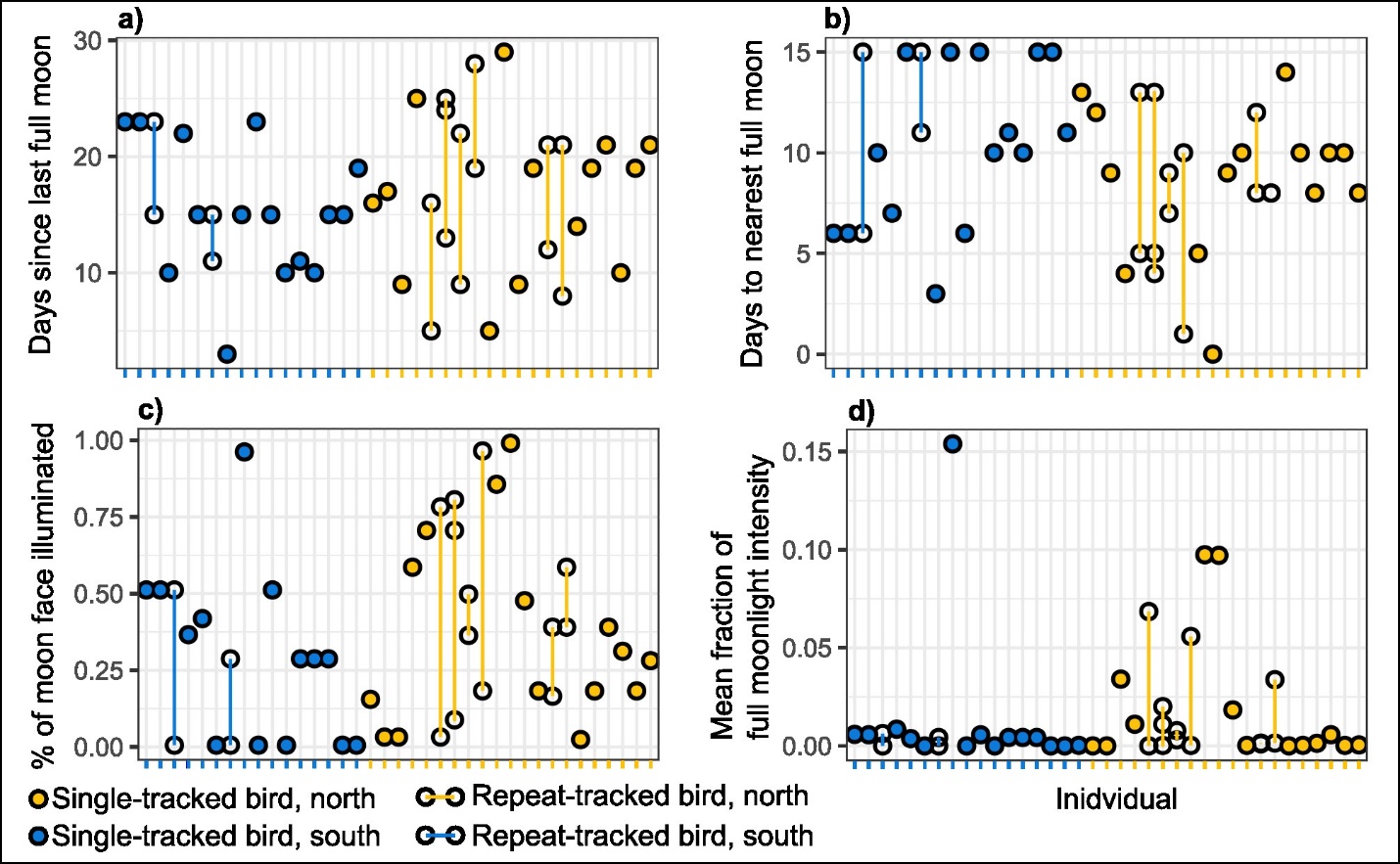


Figure S1. No evidence for individual repeatability in circalunar synchrony in Eastern Whip-poor-will autumn migration departures from two different sampling locations (north and south). Individual variation (from repeat-tracked birds) appears relatively high, compared to population-wide variation, in moon-related variables measured on the departure date for each individual: a) days elapsed since the last full moon; b) days to the nearest (past or future) full moon; c) percent of moon face illuminated, and d) mean moonlight intensity for the night. Moonlight intensity was modeled as a fraction of full moon intensity using the “moonlit” package, and is a function of disk-integrated brightness, correction for the distance to the moon, correction for atmospheric extinction, and correction for angle of incidence (Śmielak 2022).


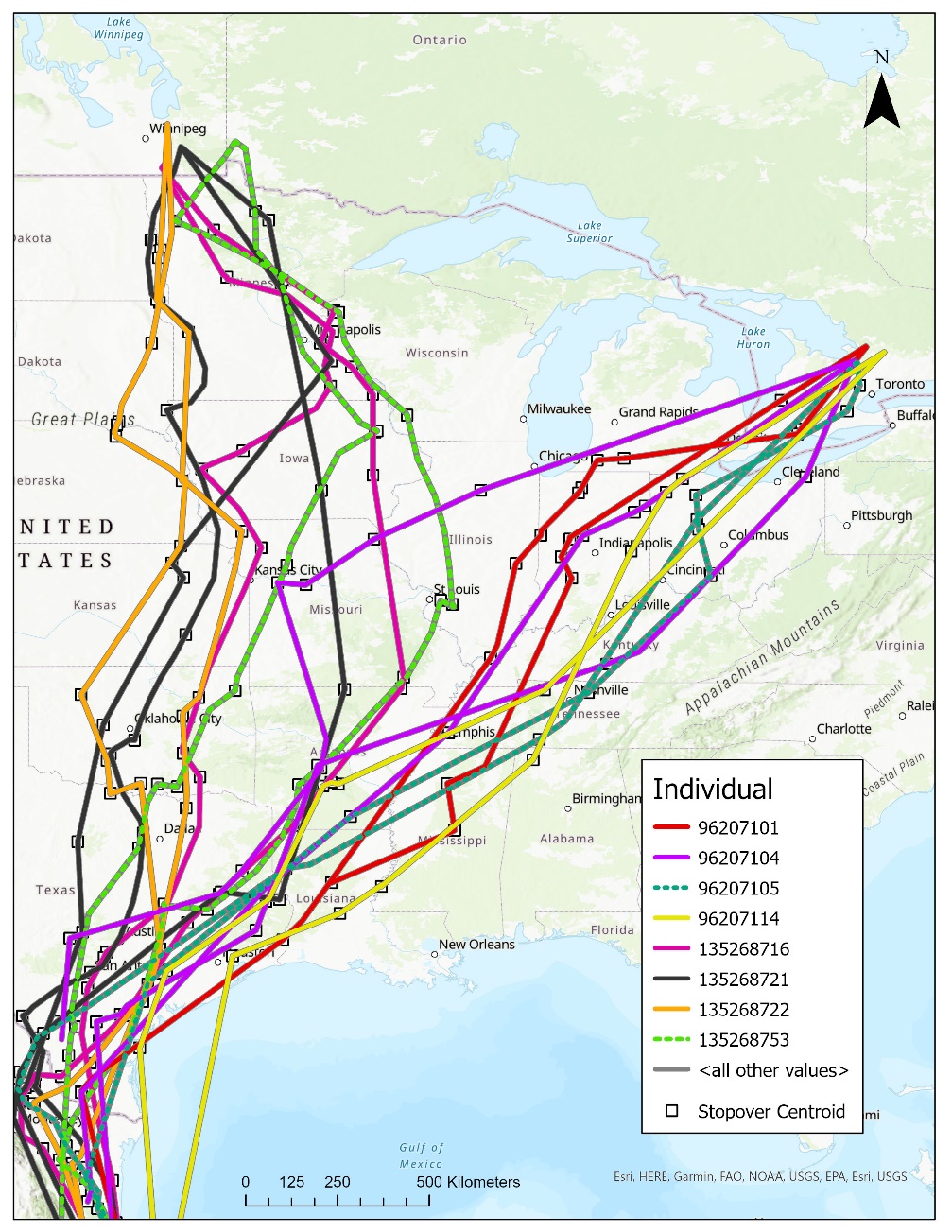


*Figure S2. Fall migration tracks through the eastern United States for a subset of repeat-tracked Eastern Whip-poor-wills. Daily stopover locations were recorded. There was no instance of repeated use of a stopover site. Two birds (96207104 and 135268721) were tracked over three years, illustrating potential for wide variation within individuals. However, most individuals appeared to follow relatively similar routes between years, compared to the population-wide variation.*

*Supplemental Table S1. Adjusted repeatabilities (intra-class correlation (ICC), with standard error (SE) and confidence intervals (CI)) for timing of Eastern Whip-poor-will migration events, after controlling for fixed effects. Only using data from repeat-tracked birds. doy = departure date day of year. area = sampling area. SeptFullMoon = date of September full moon. tracks = total number of samples collected. ind = total number of individuals tracked.*

| **Migration event** | **Model** | **tracks** | **ind** | **variation from random effect (ICC)** | **ICC SE** | **ICC CI_lower** | **ICC CI_upper** | **p** |
| --- | --- | --- | --- | --- | --- | --- | --- | --- |
| **Fall departure date** | doy ~ area*SeptFullMoon + (1\|individual) | 17 | 8 | 0.78 | 0.16 | 0.38 | 0.97 | 0.003 |
| **Lat 35 crossing** | doy ~ area*SeptFullMoon + (1\|individual) | 28 | 13 | 0.59 | 0.19 | 0.12 | 0.88 | 0.012 |
| **Lat 30 crossing** | doy ~ area*SeptFullMoon + (1\|individual) | 22 | 10 | 0.42 | 0.24 | 0 | 0.84 | 0.13 |
| **Winter arrival dates** | doy ~ area*SeptFullMoon + (1\|individual) | 10 | 5 | 0.96 | 0.1 | 0.72 | 1 | 0.006 |

*Supplemental Table S2. Conditional repeatabilities (intra-class correlation (ICC), with standard error (SE) and confidence intervals (CI)) for timing of Eastern Whip-poor-will migration events and proportion of variation explained by random and fixed effects. Only using data from repeat-tracked birds. doy = departure date day of year. area = sampling area. SeptFullMoon = date of September full moon. tracks = total number of samples collected. ind = total number of individuals tracked. Sample sizes include both single- and repeat-tracked birds.*

| Migration event | Model | tracks | ind | variation from random effect (ICC) | ICC SE | Lower ICC CI | Upper ICC CI | p | Pseudo-R^2^ (marginal) | Pseudo-R^2^ (conditional) |
| --- | --- | --- | --- | --- | --- | --- | --- | --- | --- | --- |
| Fall departure date | doy ~ area*SeptFullMoon + (1\|individual) | 17 | 8 | 0.48 | 0.2 | 0.11 | 0.84 | 0.003 | 0.38 | 0.87 |
| Lat 35°N crossing | doy ~ area*SeptFullMoon + (1\|individual) | 28 | 13 | 0.49 | 0.17 | 0.10 | 0.78 | 0.012 | 0.17 | 0.66 |
| Lat 30°N crossing | doy ~ area*SeptFullMoon + (1\|individual) | 22 | 10 | 0.32 | 0.19 | 0 | 0.69 | 0.13 | 0.23 | 0.55 |
| Winter arrival date | doy ~ area*SeptFullMoon + (1\|individual) | 10 | 5 | 0.74 | 0.23 | 0.12 | 0.96 | 0.116 | 0.22 | 0.97 |

*Supplemental Table S3. Simple slopes of the timing of migration events relative to the September full moon date for Eastern Whip-poor-wills from two distant sampling areas.*

| **Migration**  **Event** | **Sampling Area** | **Slope Estimate** | **Standard Error** | **2.5% Confidence Limit** | **97.5% Confidence Limit** | **t Value** | **p-value** |
| --- | --- | --- | --- | --- | --- | --- | --- |
| **Fall Departure** | **Northern** | -0.29 | 0.10 | -0.48 | -0.10 | -2.83 | 0.01 |
|  | **Southern** | 0.21 | 0.17 | -0.10 | 0.52 | 1.26 | 0.22 |
| **Lat 30°N Crossing** | **Northern** | -0.13 | 0.14 | -0.39 | 0.13 | -0.95 | 0.35 |
|  | **Southern** | 0.71 | 0.20 | 0.34 | 1.08 | 3.56 | 0.00 |
| **Winter Arrival** | **Northern** | -0.02 | 0.19 | -0.36 | 0.31 | -0.12 | 0.91 |
|  | **Southern** | 0.98 | 0.29 | 0.47 | 1.48 | 3.33 | 0.00 |
